# Effects of Cytokine Therapy for Treatment and Prophylaxis of Hydatidosis in Experimental Animal Model (Mice)

**Published:** 2018

**Authors:** Mahmoud RAHDAR, Abdollah RAFIEI, Rohollah VALIPOUR NOROUZI

**Affiliations:** 1. Infectious and Tropical Diseases Research Center, Health Research Institute, Ahvaz Jundishapur University of Medical Sciences, Ahvaz, Iran; 2. Dept. of Parasitology, Faculty of Medicine, Ahvaz Jundishapur University of Medical Sciences, Ahvaz, Iran

**Keywords:** Hydatid cyst, Cytokine therapy, Mice

## Abstract

**Background:**

We aimed to use of IL-12 and IFN-γ for prevention and treatment of hydatidosis in experimental animal models.

**Methods:**

This experimental study was conducted in Ahvaz City, southwest of Iran in 2017. Forty female BALB/C mice with 6–8 wk old were divided into four groups (two group test and two group control). The mice were challenged with 2000 protoscolices and a combination of IL-12 (10 μg/kg) and IFN-γ (25 μg/kg) was injected intraperitoneally daily one week at the time of challenging (prevention group) and three months after challenging (treatment group). After four months, the mice were euthanized and the number, size, and weight of the cysts were measured in all studied groups. Pathologic slides were prepared from cyst wall for pathologic changes study.

**Results:**

There issignificant reduction in number, size, and weight of cysts in test groups.

**Conclusion:**

Cytokine therapy is effective methods especially for prevention of cyst formation and to treat non-operable cases. Further study of combination of chemotherapy and cytokine therapy should be investigated.

## Introduction

Hydatidosis is one of the important zoonotic diseases in many parts of the world. Herbivorous animals act as intermediate host to complete life cycle of parasite. Human (end host) acquired infection via ingestion of eggs which passes from feces of canine family (definitive host). Hydatid cyst is a form of larva stage of *Echinococcus granulosus* a cestode worm which located in small intestinal of dogs. The disease causes economic losses in animal husbandry field and costs spending in human population for surgery and other medical procedures. The distribution of the disease is worldwide especially in area with poor sanitation, poor living condition and more closer to dogs and sheep (1, 2).

Treatment of hydatid cyst in animal husbandry is not common but in human, there are many treatment approaches. Surgery is the first choice to remove the cyst in operative cases. The surgery method has some limitation for example in patients who cannot or do not want to undergo surgery, spillage of viable protoscolices and recurrence of infection with forming secondary cyst, patients with several cysts in different organs, in no operative cases due to inaccessibility of the cyst and infections after operation (3).Albendazole and other benzimidazole carbamate drugs are the second choices for treatment of inoperative cases (4).

According to immune responses to hydatid cyst, there are four type of hydatidosis by the use of ultrasonographic finding which consists type I simple cyst with no internal architecture, type II cyst with daughter cyst at periphery, type III calcified cyst, type IV complicated cyst (ruptured cyst) (5). Variety of cellular immune responses determined the outcome of the disease. The studies show the sensitive and resistant to disease, dependent on Th1 or Th2 responses. Stimulation and activity of Th1 cytokines produce IFN-γ and IL12 and lead to protection of host and destruction of the cyst whereas the activity of Th2 cytokines response induces secretion ofIL4, IL5, IL10 and IL13 which protect the parasite from protective immune reaction and cause progression of the disease (6–8).

IL12 and IFN-γ are two major cytokine of Th1 axis. IL-12 is a heterodimeric cytokine which produced by variety of immune cells like antigen presenting cells, macrophage and lymphocytes (9, 10). IL-12 promotes and stimulates Th1 activity with production of IFN-γ and other cytokines of Th1 to proliferate of lymphocytes and natural killer cell and produce macrophage aggravation (11–13).

IFN-γ is a heterodimeric cytokine and is the only member of type II class of interferons (14). The role of the IFN-γ is very important for beginning innate and adaptive immune system reactions against viral, bacterial and parasite infections. It is produced by many immune cells such as natural killer and T cells to inhibit replication of viruses and other intracellular pathogens by activation of macrophage and increasing MHC II molecule expression (15). Administration of ginger extract to infected mice can increase the IFN-γ production and therefore is effective on hydatidosis in laboratory animal model (16). Moreover, pomegranate aquatic extract has immunomodulatory effect via TNF-a regulation and has scolicidal ability in mice hydatidosis (17).

We evaluated the therapeutic effect of Th1 cytokines (IFN-γ and IL12) on hydatidosis in experimental animal model.

## Materials and Methods

### Animals

Forty female BALB/c mice with 6–8 wk old were obtained from Laboratory Animals Breeding Center of Ahvaz Jundishapur University of Medical Sciences in 2017. Mice were divided into four groups contained 2 group control and two groups for treatment and prophylaxis assay.

All animal right ethics principles were considered according to animal right committee protocols and the study was approved by Ethics Committee of the university.

### Protoscolices preparation

Hydatid cysts were collected from infected organs of sheep in Ahvaz abattoir and transfer to parasitology laboratory of medical school. The cysts were opened under sterile condition and protoscolices were collected in sterile tubes. The protoscolices were rinsed 5 times with RPMI 1640 medium and add penicillin-streptomycin 500 unite/ml for inhibiting bacterial growth. The viability of protoscolices was tested by vital staining like eosin and more than 95% protoscolices viability were accepted for challenging BALB/c mice.

### Challenging

Two thousand live protoscolices were injected intraperitoneally in BALB/c mice. In prevention group, after challenging with protoscolices, IL12 25 (10 μg/kg) (R&D systems MA USA) (18) and interferon-gamma (25 μg/kg) (R&D systems MA USA) injected intraperitoneally for 7 consecutive days (19). Serum saline was administrated in two group control (prevention and treatment). After four months the mice were euthanized by overdosage of ketamine-xylazine and the cyst were removed from peritoneal cavity and the size, number, and weight were measured in all studied group. The pathologic section was prepared from cyst wall for pathologic examination.

### Statistical Analysis

Fisher exact test and chi-square were used for statistical analyzing and *P.*value <0.05 was considered for significant differences. The used software was SPSS (ver.21, Chicago, IL, USA)

## Results

### Comparison of number of Hydatid cysts in studied groups

**Fig. 1:**
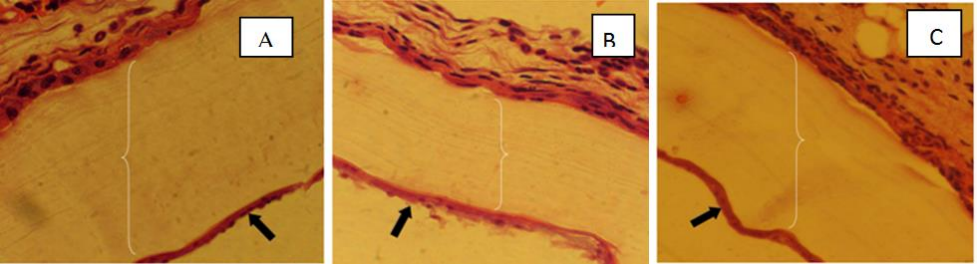
Histological Changes of Cysts in Prophylaxis (A), Treatment (B) and Control (C) Groups of Balb/c Mice

The average number of cyst in prevention and control groups were 2 and 1551, respectively; there are significant differences between prevention and control group for number of Hydatid cyst (*P*<0.05). The results of treatment and control groups also showed significant differences between two groups which the number of cysts were 5.7 and 15 respectively (*P*<0.01). The results are presented in Table 1 and Fig. 1.

**Table 1:** The number of Hydatid cyst in treatment, prevention and control groups

***Group***	***Number of mice***	***Mean***	***Efficacy rate (%)***	***Standard deviation***
IFN γ + IL12 (Prevention)	9	2.000	87	3.041
IFN γ + IL12 (Treatment)	9	5.7	63	3.708
Control (prevention)	8	15	0	4.472
Control (Treatment)	9	15.33	0	3.708

### Comparison of size of Hydatid cysts in studied groups

The analysis of cysts size indicated that there are significantly differences between prevention, treatment group and control groups (*P*<0.001) (Table 2).

**Table 2:** The size of Hydatid cyst in test and control groups of Balb/cmice

***Group***	***Number of mice***	***Mean***	***Efficacy rate (%)***	***Standard deviation***
IFN γ + IL12 (Prevention)	9	3.8	40	2.7
IFN γ + IL12 (Treatment)	9	4.9	18.3	3
Control (prevention)	8	6.3	0	2.4
Control (Treatment)	9	6	0	2.4

### Comparison of weight of Hydatid cysts in studied groups

**Fig. 2:**
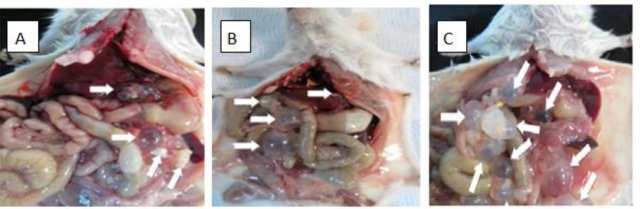
The hydatid cyst in prevention (A) treatment (B) by cytokines and control (C) groups

There are significant differences between prevention, treatment and control groups in weight of cysts (*P*<0.05) (Table 3). Fig. 2 shows the cyst in BALB/c mice in studied groups.

**Table 3:** The weight of Hydatid cyst in treatment, prevention and control groups

***Group***	***Number of mice***	***Mean***	***Efficacy rate (%)***	***Standard deviation***
IFN γ + IL12 (Prevention)	9	0.13	93.5	0.29
IFN γ + IL12 (Treatment)	9	0.46	81.5	0.72
Control (prevention)	8	2	0	0.68
Control (Treatment)	9	2.5	0	1.18

## Discussion

Using surgery and chemotherapy methods with benzimidazole family drugs is currently applied for treating human hydatidosis. Some adverse effects of two methods lead us to select the other alternative choice. The advantage of cytokine therapy is confirmed and applied for treatment or prophylaxis of some infectious diseases. The benefit of cytokine therapy in human tuberculosis was shown (20). The use of proinflammatory cytokine inhibitors may prove beneficial for tumor therapy (21). The role of Th1/Th2 in viral hepatitis (22) was evaluated. In this condition Th1/Th2 axis determined the clinical outcome (23, 24).

The benefit of cytokine therapy in visceral leishmaniasis (25, 26), asthma (27), bacterial infection (28), inflammatory diseases (29) are showed. In progressive alveolar hydatidosis cyst cases, the production and activity of Th2 cytokines such as IL-10, IL-5 and IL-2 is reported (30).

The role of Th1 cytokines in initiation of protective immune responses against pathogens agents such as viral, bacterial and parasite is well known. Two predominant Th1 cytokines are IL-12 and IFN-γ5 They stimulate and increase macrophage and NK cells population to destroy pathogen agents. The advantage of IL-12 injection to treat *Leishmania major* infection in BALB/c mice was presented. The treatment of infected BALB/c mice by IL-12 one week after challenging with *L. major* showed a significant healing due to promotion of Th1 and suppression of Th2 cytokines (31). The protective role of IL-12 has executed via induction of IFN γ production (32). The use of IL-12 in infected BALB/c mice with *Trypanosoma cruzi* cause a significant reduction of parasites burden by increasing IFN- γ and TNF-α levels (33). Administration of IL-12 to *Schistosoma mansoni* infected mice caused reduction of egg-induced pathologic changes by inhibition of granuloma formation and increasing of Th1 cytokines (34).

The present study conducted to evaluate the effect of Th1 cytokines for treating and preventing monolocular hydatid cyst in animal model. To evaluate prophylaxis effect of cytokine therapy in hydatid cyst infection, we administrated a combination of IL-12 and IFN- γ after challenging of 2000 protoscolices intraperitoneal injection. The efficacy of combination of IL-12 and IFN- γ to reduce number, weight, and size of the cyst was 87%, 93.5%, and 40% respectively when administrated at the time of challenge. The protective role of TNF-α in alveolar echinococcosis was investigated. The growth of alveolar echinococcosis was compared in two group of the BALB/c mice included LT- α TNF- α +/+ and LT- α TNF- α −/− allele. The burden of the parasites in LT- α TNF- α−/− mice were very higher than wild-type mice (LT-α TNF- α +/+ allele). The parasites load in heterozygous mice was intermediate between LT- α TNF- α −/− and wild-type mice (35). Decreasing of IFN- γ in late stage of alveolar echinococcosis infection in mice is associated with high parasite burden (36). The treatment of experimental model of alveolar echinococcosis with IFN- γ (1–5 μg twice in week for three weeks) reduced parasite burden in liver and it was suggested that administration of IFN- γ with appropriated drug is more effective (37).

The effect of IL-12 injection to *E. multilocularis*-infected mice (C57BL/6 J) was evaluated. The administration of IL-12 at the challenging time reduced parasite burden and inhibit development of disease. The combination of IL-12 and albendazole should be considered for treatment and prevention of alveolar echinococcosis in human (18).

We evaluated the efficacy of cytokine therapy for treatment of progressive hydatidosis condition too. A combination of IL-12 and IFN- γ administration to echinococcosis-infected mice three months after challenging, reduced number, weight and size of cyst 63%, 81.5%, and 18.3% respectively. Although the administration of cytokines after establishing infection significantly was effective, the use of cytokine therapy was more effective for prevention purpose, agreement with Emery study (18).

## Conclusion

Using Th1 cytokines could be effective on treatment and prevention of hydatidosis in human especially for prevention of secondary hydatid cyst development. Further studies are needed to evaluate treatment and prevention effect of cytokine and chemotherapy combination for hydatidosis.
